# Photocatalytic Decomposition of Carmoisine and Crystal Violet by Ho-Doped TiO_2_ Sol-Gel Powders

**DOI:** 10.3390/ma19010017

**Published:** 2025-12-20

**Authors:** Nina Kaneva, Stefani Petrova, Albena Bachvarova-Nedelcheva

**Affiliations:** 1 Laboratory of Nanoparticle Science and Technology, Department of General and Inorganic Chemistry, Faculty of Chemistry and Pharmacy, University of Sofia, 1 James Bourchier Blvd., 1164 Sofia, Bulgaria; 2 Institute of General and Inorganic Chemistry, Bulgarian Academy of Sciences, Acad. G. Bonchev, Str., bl. 11, 1113 Sofia, Bulgaria; stefany@svr.igic.bas.bg; 3 National Centre of Excellence Mechatronics and Clean Technologies, 8 bul., Kl. Ohridski, 1756 Sofia, Bulgaria

**Keywords:** photocatalysis, anionic dye, cationic dye, sol–gel

## Abstract

This study explores the sol–gel synthesis, structural characterization, and photocatalytic performance of Ho^3+^-doped TiO_2_ nanopowders at two dopant levels (0.5 and 2 mol%). Transparent, homogeneous gels were prepared using titanium (IV) butoxide and holmium (III) nitrate pentahydrate in ethanol, followed by drying and optional annealing at 500 °C. X-ray diffraction confirmed anatase TiO_2_ as the dominant crystalline phase, with Ho incorporation suppressing crystal growth and yielding smaller crystallite sizes than undoped TiO_2_. FT-IR and UV-Vis spectroscopy verified complete hydrolysis–condensation during gel formation, while diffuse reflectance spectra revealed a red-shifted absorption edge, indicating reduced band gaps. SEM analysis showed nanoscale particles with agglomeration, which intensified after annealing. Photocatalytic activity was tested under UV irradiation using Crystal Violet (anionic dye) and Carmoisine (cationic dye). Annealed Ho-doped powders exhibited markedly higher degradation rates, with the 2 mol% sample achieving the greatest efficiency, particularly against Crystal Violet. These findings demonstrate that Ho^3+^ doping enhances TiO_2_’s UV-driven photocatalytic activity by tailoring its structural and optical properties.

## 1. Introduction

The most fundamental human need for health and wellbeing is access to clean water. Rapid urbanization, population expansion, and increased water demands from the energy, industrial, and agricultural sectors are all contributing to the rise in water demand.

Pollution of water resources is a growing concern because of the hazardous nature of many contaminants. Some of the most prominent industrial pollutants are organic dye effluents, which pose a serious ecological and health hazard due to their carcinogenicity and toxicity for the aquatic life [1]. The structural diversity of dyes makes them particularly challenging to remove with conventional purification methods. While techniques such as adsorption, coagulation, and membrane filtration [2,3] have been widely applied, their selectivity and limited efficiency highlight the urgent need for more versatile approaches.

Advanced oxidation processes, such as heterogeneous photocatalysis, have become a promising solution to the rising problem because of their cost effectiveness and environmentally friendly approach [4]. However, photocatalytic efficiency is strongly influenced by both dye chemistry and catalyst surface properties, which means that a single unmodified semiconductor often performs inconsistently across different pollutants. Such selectivity has been reported in several studies where photocatalyst performance varied depending on dye charge and molecular structure. This has led to the development of modification strategies, such as metal or rare-earth doping of popular semiconductors like ZnO, TiO_2_, g-C_3_N_4_, etc. [4], that can enhance charge separation, extend light absorption, and improve overall catalytic activity. For example, Verma et al. [5], report that hydrothermal Au-doped ZnO nanorods show better photocatalytic activity against Methylene Blue, a cationic dye with smaller molecular weight [6], compared to Methyl Orange (anionic azo-dye with relatively bigger molecular weight) [7] and Rhodamine B (a zwitterion in aqueous solution) [8]. Furjadfar et al.’s study [9] reports that carbon dot-encapsulated TiO_2_ demonstrates better photocatalytic efficiency against cationic dyes in higher pH conditions, while anionic dyes were better removed under lower pH conditions. In Rajagopal et al.’s research paper [10], H_2_O_2_-assisted TiO_2_ successfully destroyed Methylene Blue, while the degradation process of Acid Violet (an anionic dye) was much slower. The literature study clearly shows that there is a prominent need for a photocatalyst that can successfully remove various effluents under normal conditions.

Beyond noble metal or transition metal modifications, rare-earth doping has emerged as a particularly promising strategy due to the unique electronic configurations of lanthanides, which facilitate charge separation and visible light absorption [11]. Scientific interest has shifted towards incorporating Lanthanide (LN) ions into popular semiconductors (as mentioned above) and evaluating their effect on the degradation of various pollutants. Karakaya et al.’s group has verified the improvement of photocatalytic properties in Gd and Ce co-doped ZnO [12]. In particular, a lot of research is focused on improving TiO_2_ nanoparticles (NPs) which are widely used in photocatalysis because of their tremendous properties such as multifunctionality, low toxicity, and high chemical stability [13]. For example, Hassan et al.’s group has demonstrated enhancement in the photocatalytic performance of titania after doping with La, Ce, and Nd [14]. Similarly, nonhydrolytic sol–gel-obtained La-modified titanium oxide has been shown to improve the degradation of Paracetamol [15]. A Gd-modified titanium dioxide was prepared from leaf extracts and has shown almost complete decomposition of Tetracycline hydrochloride [16]. Although some studies have explored Ho^3+^-doped TiO_2_ [17,18,19], most focus on structural or optical properties rather than systematic evaluation against both cationic and anionic dyes under comparable conditions. Moreover, sol–gel synthesized Ho^3+^-TiO_2_ remains underexplored in this context.

Our team has extensive experience in the synthesis of titania-based materials, doped or composites [20,21,22]. Based on our previous work, we recognize that many aspects regarding the influence of dopants on the structural, optical, and photocatalytic properties of TiO_2_ remain insufficiently understood. Therefore, we continue to investigate these effects in order to gain a deeper understanding and establish clearer correlations between dopant type, concentration, and material performance.

The objective of this study is to investigate the effect of Ho^3+^ doping on the physicochemical properties and photocatalytic performance of sol–gel-synthesized TiO_2_. The photocatalytic activity was evaluated against two structurally distinct dyes—Carmoisine (an anionic azo dye consisting of two naphthalene subunits, also known as Azorubine or E122) and Crystal Violet (a cationic triphenylmethane dye)—to assess the potential of Ho^3+^ modification in achieving broad-spectrum degradation of organic pollutants.

Despite the extensive research on modified TiO_2_ photocatalysts, a clear knowledge gap remains regarding the systematic evaluation of rare-earth-doped TiO_2_ synthesized under comparable conditions and tested against structurally contrasting pollutants. In particular, Ho^3+^ doping has been investigated mainly for its structural or optical effects, with very limited studies examining its influence on photocatalytic selectivity toward anionic versus cationic dyes. Moreover, sol–gel synthesized Ho^3+^-TiO_2_ remains largely unexplored, even though the sol–gel route allows superior dopant dispersion, controlled nanostructure formation, and tunable surface properties.

## 2. Materials and Methods

### 2.1. Sample Preparation

To explore the effect of Ho^3+^ concentration on TiO_2_ structure and performance, doping levels of 0.5 mol% and 2 mol% were chosen. These were selected based on prior studies indicating that rare-earth (lanthanide) ions typically exhibit optimal photocatalytic enhancements at relatively low loadings, often below ~1–3 mol%, while higher concentrations can lead to dopant clustering, defect formation, or recombination centers that degrade performance [23].

**Figure 1:**
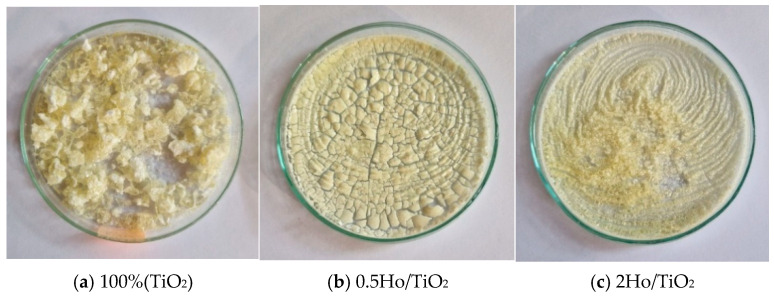
Visual observations of the as-prepared gels.

Therefore, compositions containing different holmium content were synthesized: 0.5Ho/TiO_2_ and 2Ho/TiO_2_ (mol%). Ti(IV) n-butoxide (Ti(C_4_H_9_O)_4_, (Fluka Chemie AG, Darmstadt, Germany), holmium (III) nitrate pentahydrate, Merck, Rahway, NJ, USA), and ethanol (C_2_H_5_OH, Merck) were used as precursors. For the synthesis of the samples, two solutions were prepared with continuous stirring for 5–10 min, with different precursor concentrations. The precursors were separately dissolved in ethanol at a 1:1 ratio. Titanium (IV) butoxide was then simultaneously introduced into the solution containing holmium nitrate. The pH of the resulting solutions was measured to be between 4 and 5. Gelation occurred instantly upon mixing, producing homogeneous gels. These gels were aged at room temperature (23 ± 2 °C) for one week. Following aging, a portion of each sample was annealed at 500 °C for 2 h to examine phase transformations of the prepared gels (Figure 1). The resulting data were compared with those obtained for pure TiO_2_ synthesized from the Ti (IV) butoxide precursor [24]. The investigated samples 0.5 mol% Ho-doped TiO_2_ and 2 mol% Ho-doped TiO_2_ were denoted as 0.5Ho/TiO_2_ and 2Ho/TiO_2_, respectively.

### 2.2. Characterization Techniques

According to the X-ray diffraction method (XRD), the produced materials’ crystalline phases were identified. A Bruker D8 Advance X-ray diffractometer (Bruker, Bremen, Germany) equipped with Cu Kα (α = 1.5418 Å) was used to conduct the analysis. Step-scanning mode (Δ2θ = 0.05°) was used to produce the patterns throughout an angular range of 10–80°. The average crystallite size (D) of the samples was estimated using the Scherrer equation:

(1) $$D\;=\frac{K\lambda }{\beta \cos\theta },$$

where K is the shape factor (0.9), λ is the X-ray wavelength (0.15418 nm), β is the full width at half maximum (FWHM) of the diffraction peak (in radians), and θ is the Bragg angle. The crystallinity was calculated following the formula:

(2) $$\%\;\mathrm{Crystallinity}\;=\;\frac{\mathrm{Area}\;\mathrm{of}\;\mathrm{crystalline}\;\mathrm{peaks}}{\mathrm{Area}\;\mathrm{of}\;\mathrm{crystalline}\;\mathrm{and}\;\mathrm{amorphous}\;\mathrm{peaks}}100,$$

The microstrain (ε) was calculated using the relation

(3) ε=β/(4tanθ),

which reflects the lattice distortion within the crystal domains. The dislocation density (*δ*), representing the number of dislocations per unit area of the crystal, was estimated as

(4) $$\delta =1/D^{2}$$

Optical absorption spectra of the powdered samples were recorded in the 200–800 nm range using a UV–Vis diffuse reflectance spectrophotometer (Evolution 300, Thermo Electron Corporation, Madison, WI, USA), with magnesium oxide employed as the reflectance standard baseline. Tauc’s equation was used to estimate the band gap energy:

(5) $$\alpha h\nu ={A(h\nu -E_{g})}^{\frac{n}{2}}$$

In this equation, A is a constant that is independent of hν, *E_g_* is the semiconductor band gap, and *n* is dependent on the kind of transition.

Infrared spectra were recorded in the 1600–400 cm^−1^ range using the KBr pellet technique on a Nicolet 320 FTIR spectrometer (Thermo Fisher Scientific, Madison, WI, USA) with 64 scans and a resolution of ±1 cm^−1^. Sample morphology and microstructure were examined using a JSM-5510 scanning electron microscope (JEOL, Tokyo, Japan) operated at an acceleration voltage of 10 kV. Prior to imaging, the samples were coated with gold using a JFC-1200 fine coater (JEOL). Additional characterization was performed with a Zeiss Evo 15 microscope (Zeiss, Oberkochen, Germany) equipped with energy-dispersive X-ray spectroscopy (EDS). The specific surface areas of catalysts heat-treated at 500 °C were determined by low-temperature (77.4 K) nitrogen adsorption using a NOVA 1200e surface area and pore analyzer (Quantachrome, Boynton Beach, FL, USA) at relative pressures (p/p_0_ = 0.1–0.3), applying the BET equation. The samples’ room temperature photoluminescence (PL) was measured using a Varian Cary Eclipse Fluorescence UV-vis spectrophotometer set to 320 nm as the excitation wavelength.

### 2.3. Photocatalytic Activity Experiments

Carmoisine and Crystal Violet (dyes for coloring confections, sugar glazes, and drinks, Dolce Maestro, Sofia, Bulgaria) were used as model dye pollutants to assess the photocatalytic breakdown capacities of the synthesized and examined samples under UV light irradiation. The Evolution 300 UV-VIS spectrophotometer (Thermo Scientific, Waltham, MA, USA, 50–60 Hz, 150 VA) with a wavelength range of 300 to 600 nm, was used to measure the optical absorption spectra and the pollutant concentration following irradiation. The organic pollutant’s original concentration was 7.5 parts per million. An electromagnetic stirrer and a 200 mL glass cylindrical vessel with a magnetic stirrer and a UV lamp above it were utilized for the photocatalytic process. There were fifteen minutes of darkness. Using UV-vis absorption spectroscopy, the degradation of the pollutant during the photocatalytic process was examined using aliquot samples (2 mL) collected at predetermined intervals. Prior to being measured using the spectrophotometer, the aliquot samples of the photocatalysis with powders were run through a microfilter to eliminate the suspension. The intensity of the dye’s absorption spectra in the solution decreased over the irradiation period, and the following formula was used to determine the degree of degradation:

(6) $$D\%=\frac{(C_{0}\;-\;C)}{C_{0}}\times 100,$$

where C represents the sample concentration and C_0_ is the initial concentration.

This enables us to assess the degree of dye decomposition. Two series of studies were carried out: non-annealed gels and powders annealed at 500 degrees. Every photocatalytic test was conducted using distilled water at room temperature (23 ± 2 °C) with a continuous stirring speed of 500 rpm.

Furthermore, no indication of direct dye degradation was found in trials carried out in the absence of a photocatalyst (photolysis). To evaluate reproducibility, every degradation experiment was carried out in triplicate.

## 3. Results and Discussion

### 3.1. XRD Analysis

**Figure 2:**
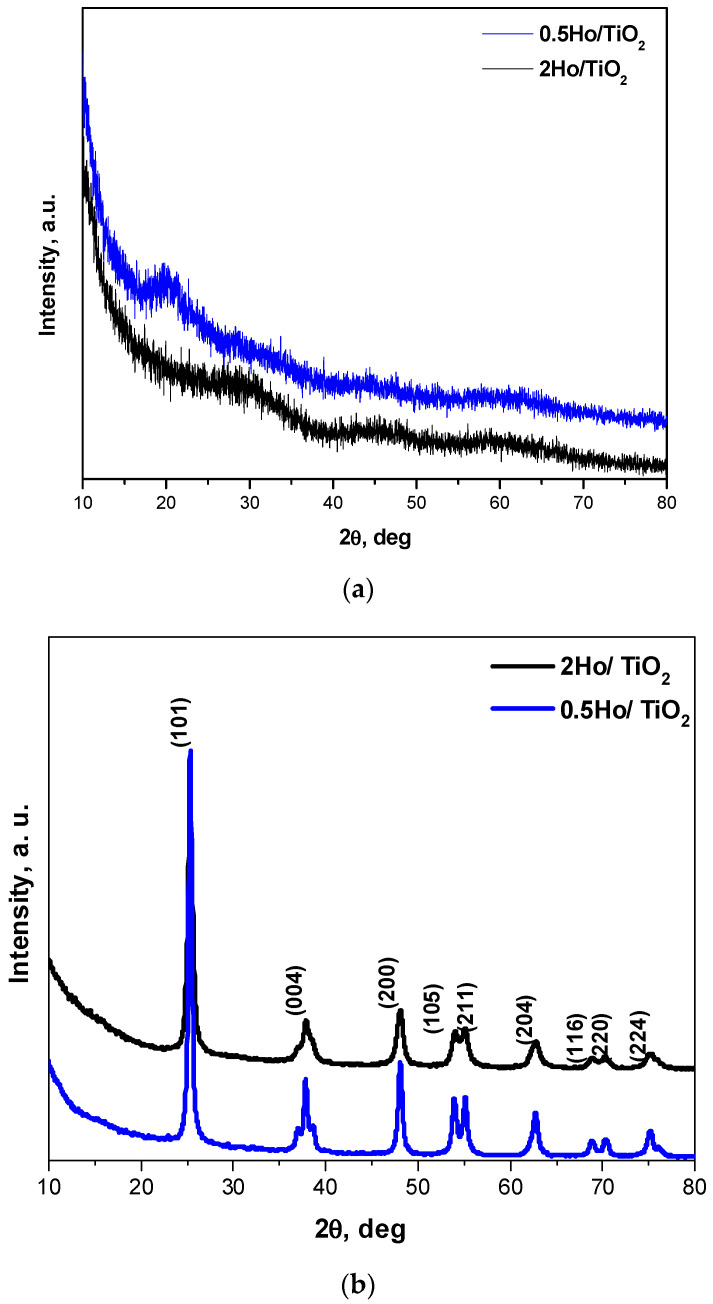
X-ray diffraction patterns of Ho doped TiO_2_ samples: gels (**a**) and annealed at 500 °C samples (**b**).

The phase composition of the Ho^3+^-doped samples, both annealed and non-annealed, was examined via XRD (Figure 2). The diffractograms of the heat-treated samples display sharp and intense reflections, characteristic of a highly crystalline structure, which can be indexed to the anatase phase of TiO_2_ (JCPDS 78-248). No additional peaks attributable to Ho_2_O_3_ or other Ho-containing phases are observed, suggesting that Ho^3+^ ions are either successfully incorporated into the TiO_2_ lattice or dispersed below the detection limit of XRD. The incorporation of Ho^3+^ ions into the anatase Ti^4+^ sites introduces lattice distortions due to the ionic radius mismatch (Ho^3+^ = 1.015 Å; Ti^4+^ = 0.605 Å for six-fold coordination) [25]. This size difference generates local strain, which suppresses the growth of grains during annealing and leads to the observed decrease in crystallite size [26]. The peaks become broader in width and weaker in intensity with the increase in Ho content, which is in good agreement with previously reported data [27]. This broadening indicates that Ho^3+^ doping inhibits the crystallization process and reduces the size of the nanocrystals.

Quantitative analysis confirmed a slight decrease in both crystallinity and crystallite size with increasing dopant concentration: the crystallinity decreased from 63% (0.5Ho/TiO_2_) to 57% (2Ho/TiO_2_), while the average crystallite size, calculated using the Scherrer equation, decreased from 15 nm to 13 nm, respectively. The 2% Ho sample shows higher microstrain (0.0127 vs. 0.00892) and higher dislocation density (0.00569 vs. 0.00472 nm^−2^) than the 0.5% Ho one. This is consistent with increased lattice distortion and defect density at higher dopant content [14,28]. These results corroborate that Ho incorporation leads to a minor disruption of the TiO_2_ lattice, introducing lattice strain and defects that hinder crystal growth. In contrast, the non-annealed gel-derived powders exhibit broad humps without distinct diffraction peaks, indicative of an amorphous structure. The XRD pattern of undoped TiO_2_ is not presented here, as it has been reported previously by the authors [24].

The specific surface area (SSA) of the 2Ho/TiO_2_ sample was measured and found to be 3 m^2^/g. For the heat-treated sample, the SSA was 7 m^2^/g. As determined by the BET analysis, the larger specific surface of the heat-treated catalyst is one of the necessary conditions for its higher catalytic efficiency compared to the non-heat-treated samples.

### 3.2. SEM Surface Morphology Results

**Figure 3:**
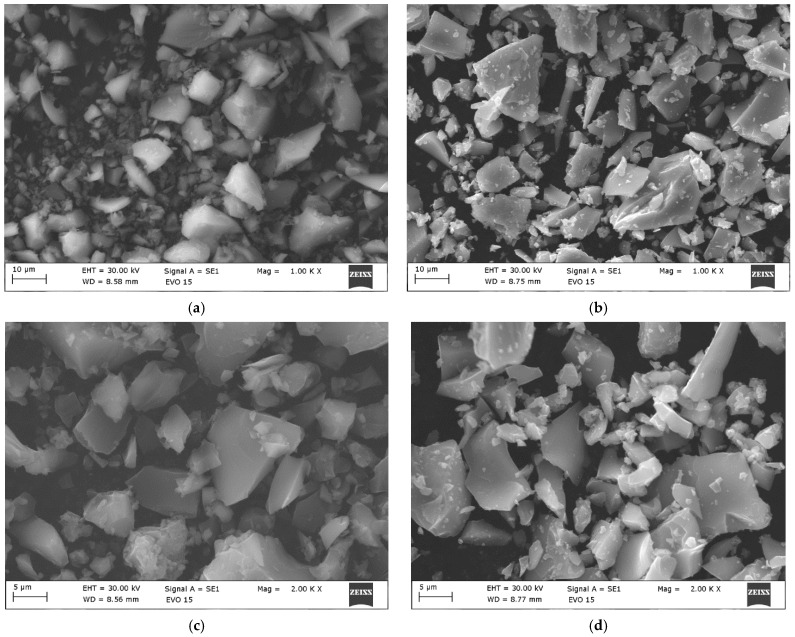
SEM images at different magnifications of (**a**,**c**) nonannealed 2Ho/TiO_2_ and (**b**,**d**) annealed at 500 °C 2Ho/TiO_2_.

The microstructure of the 2Ho/TiO_2_ was investigated using Scanning Electron Microscopy (SEM). The representative images at different magnifications are presented in Figure 3a,c, revealing the morphological characteristics of the powder. Only the SEM observations of the 2Ho/TiO_2_ sample are presented in this study, as it exhibited superior photocatalytic activity compared to the other one. At low magnification (1000×), the SEM micrograph shows presence of particles with a predominantly plate-like morphology. These particles vary in size from approximately 10 µm to over 50 µm. The surfaces of the observed particles appear relatively smooth, but many are covered with smaller ones that are clustered around the larger ones. This particle distribution indicates a multi-phase microstructure, which is consistent with the XRD results. At higher magnification (5000×), as shown in Figure 3c, larger particles (~15–20 µm) are observed with numerous fine granular structures adhered to their surface. These fine particles appear to be <1 µm in size. The absence of significant porosity on the particles surface suggests good densification.

Figure 3b,d shows SEM micrographs of Ho-doped TiO_2_ powders annealed at 500 °C. As it is seen from the figure, during the annealing the morphology of the particles did not change and powders were higher agglomerated. At lower magnification (1000×) (Figure 3b), the sample exhibits an agglomerated structure composed of irregularly shaped particles. At higher magnification (5000×) (Figure 3d), individual crystallites become more distinguishable, revealing plate-like morphology. The particles exhibit significant agglomeration, forming loosely packed clusters ranging up to 20–30 µm in size. This agglomeration is typical for oxide nanoparticles synthesized via sol–gel. A similar result for SEM analysis was reported by [29].

### 3.3. Ho/TiO_2_ Elemental Mapping and EDS Analysis

**Figure 4:**
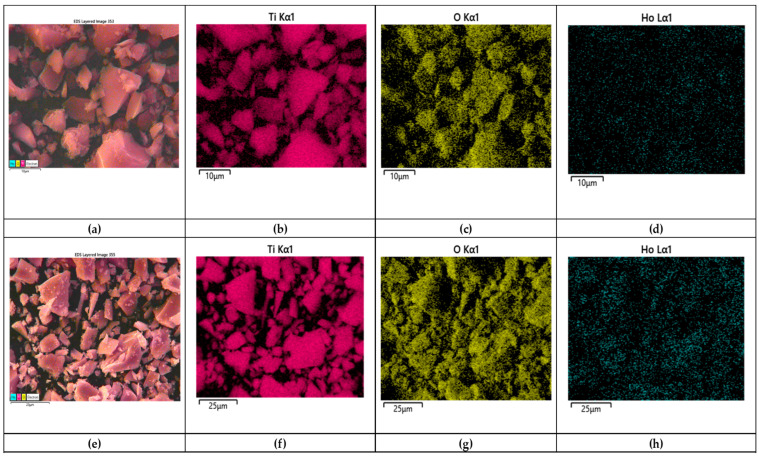
SEM mapping of 2% Ho doped TiO_2_ (**a**–**d**) gel and heat-treated sample (**e**,**f**); composition map of Ti (**b**,**e**); composition map of O (**c**,**f**); composition map of Ho (**d**,**g**,**h**).

**Figure 5:**
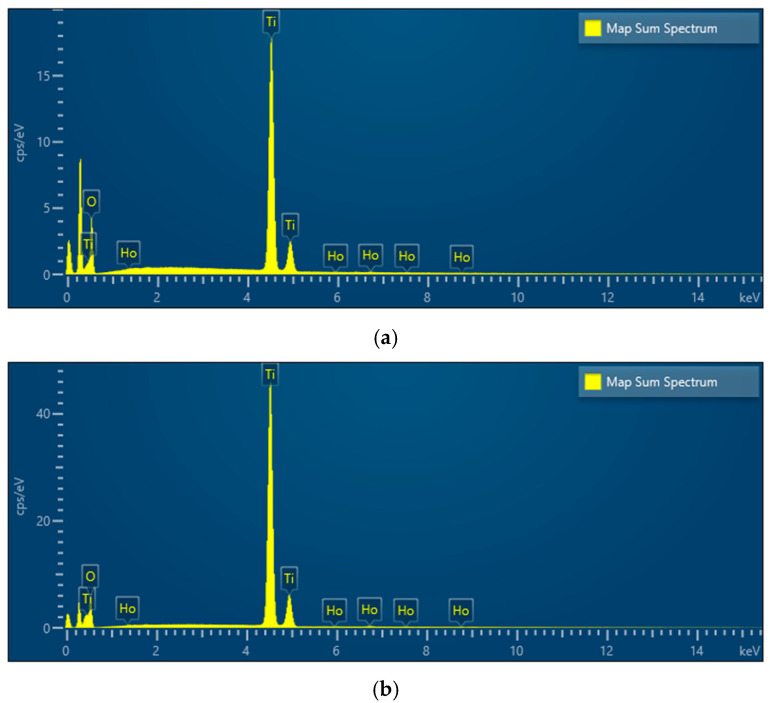
EDS of 2% Ho doped TiO_2_ indicating elemental composition—gel (**a**) and heated at 500 °C sample (**b**).

SEM elemental mapping of Ho-doped TiO_2_ gel heat-treated at 500 °C is shown in Figure 4a–h with the corresponding elemental composition by EDS in Figure 5a,b. The mapping shows that all elements are well distributed across the surface of the sample. The EDS detected the composition of titanium (Ti), oxygen (O), and holmium (Ho) elements. No carbon (C) was detected after the annealing for 2 h exposure time. The obtained data correlates with those obtained by other authors [17,30].

### 3.4. IR Spectral Data

**Figure 6:**
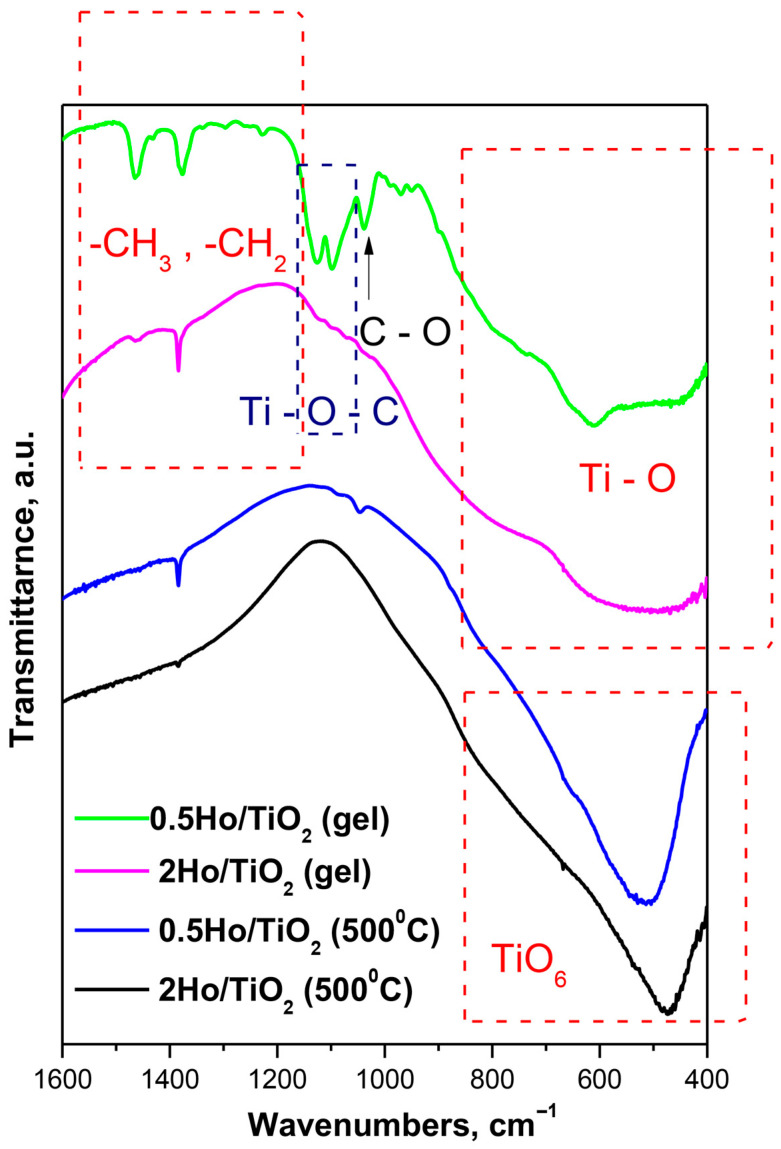
IR spectra of the gels and heat-treated at 500 °C Ho-doped TiO_2_ samples.

The structural investigations of the as-prepared gels and heat-treated at 500 °C Ho/TiO_2_ powders were investigated by means of IR spectroscopy, and the stretching vibrations of the inorganic building units in the range of 1600–400 cm^−1^ (Figure 6) have been considered. Looking at the spectra, there is a significant difference between the gels and the annealed samples. The IR spectra of the gels also differs from that of 0.5Ho/TiO_2_, and it is very similar to the Ti buthoxide spectrum which has been discussed in details in our previous papers [31]. The IR spectrum of 2Ho/TiO_2_ exhibited the following bands at 800, 590, and 470 cm^−1^ that could be related to the Ti-O vibrations [15]. Several bands could be distinguished in the IR spectrum of the gel 2Ho/TiO_2_ above 1000 cm^−1^—1470, 1400, 1070, and 1130 cm^−1^. It is well known that the bands located between 1500 and 1300 cm^−1^ are assigned to the bending vibrations of CH_3_ and CH_2_ groups. The band at 1130 cm^−1^ is characteristic for the stretching vibrations of Ti-O-C, while those at 1070 cm^−1^ are assigned to the vibrations of terminal and bridging C-O bonds in butoxy ligands [32]. The IR spectra of heat treated at 500 °C samples are dominated by the bands in the low frequency region 800–470 cm^−1^ which could be ascribed to the TiO_6_ polyhedra. It has been found that the characteristic vibrations of Ho-O are between 600 and 500 cm^−1^ [33]. Having in mind the spectra obtained by us, it is difficult to distinguish the characteristic bands of the Ti-O and Ho-O structural groups.

### 3.5. UV-Visible Spectroscopic Analysis Results

**Figure 7:**
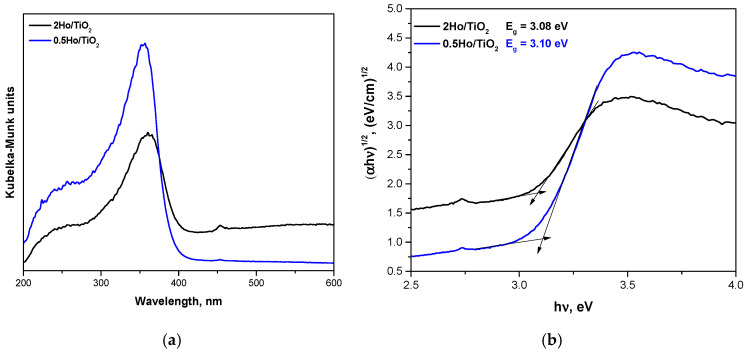
UV-Vis spectra of the investigated samples (**a**) and band gap energy (*E_g_*) determination using Tauc equation (**b**).

The UV-Vis diffuse reflectance spectra (Figure 7), converted via the Kubelka–Munk function, reveal a systematic red shift in the absorption edge with increasing Ho^3+^ concentration. Tauc analysis (indirect transition, *n* = 1/2) yields optical band gap energies of 3.25 eV for pure TiO_2_, 3.10 eV for 0.5Ho/TiO_2_, and 3.08 eV for 2Ho/TiO_2_, corresponding to a reduction of ≈0.17 eV relative to undoped TiO_2_. Both direct and indirect Tauc models have been evaluated for the estimation of the optical band gap. When applying linear fitting to the respective regions, we found that the indirect transition model provides a significantly better linear correlation. The absorption edge values of TiO_2_, 0.5Ho/TiO_2_, and 2Ho/TiO_2_ are 380.44 nm, 400 nm, and 402.5 nm, respectively. The observed red shift upon Ho incorporation suggests the formation of Ho-related localized states and defect levels (such as oxygen vacancies) that act as charge-compensating centers and arise from lattice distortion during doping. This behavior is consistent with previous reports on Ho^3+^-doped TiO_2_, where similar narrowing of the band gap and enhanced visible light absorption were observed [17].

The characteristic absorption band of TiO_2_ around 320 nm originates from the O 2p → Ti 3d charge-transfer transition within TiO_6_ octahedra formed during the hydrolysis-condensation of Ti species [34]. Upon Ho^3+^ doping, the overall absorption intensity increases relative to undoped TiO_2_, indicating improved light-harvesting efficiency due to the introduction of 4f-related Ho states and enhanced charge-transfer interactions between TiO_6_ units. However, a slight decrease in intensity is observed for 2Ho/TiO_2_ compared to 0.5Ho/TiO_2_, which can be attributed to increased lattice disorder, dopant clustering, and the formation of additional defect sites at higher Ho concentrations [35,36]. These factors likely promote nonradiative recombination and partial disruption of the TiO_6_ network, consistent with prior observations in rare-earth-doped TiO_2_ systems [35,37]. Doping with Ho^3+^ introduces shallow defect states, oxygen vacancies, and lattice strain due to its larger ionic radius, while its localized 4f orbitals remain highly shielded and do not significantly hybridize with the TiO_2_ valence or conduction bands [38]. These effects lead to minor numerical changes in the band gap but substantial improvements in charge separation and carrier lifetime.

The very similar band gap values obtained for 0.5Ho/TiO_2_ and 2Ho/TiO_2_ (3.10 and 3.08 eV, respectively) likely reflect small experimental deviations rather than a significant electronic difference between the two samples.

The conduction band (CB) and valence band (VB) potentials were calculated using the following relations:

(7) $$E_{CB}\;=\;\chi \;-\;4.5\;-\;0.5E_{g}\;$$

(8) $$E_{VB}=E_{CB}+E_{g}\;,\;$$

where *χ* is the absolute electronegativity of the semiconductor. The calculated potentials (Table 1) show that the CB edge becomes slightly less negative with Ho^3+^ incorporation (from −0.313 eV for TiO_2_ to −0.238 eV for 0.5Ho/TiO_2_ and −0.230 eV for 2Ho/TiO_2_), while the VB edge correspondingly shifts from +2.93 eV to +2.86 eV and +2.85 eV, respectively. These small variations indicate that Ho doping does not significantly alter the band structure of TiO_2_. Nevertheless, both Ho-doped samples exhibit enhanced light absorption and improved charge separation efficiency compared to pure TiO_2_, resulting in higher photocatalytic activity.

The conduction band positions of the Ho-doped samples (−0.23 to −0.24 eV vs. Normal Hydrogen Electrode) remain sufficiently negative to promote the reduction of O_2_ to·O_2_^−^ radicals, while their valence band potentials (+2.85 to +2.86 eV) provide enough oxidative power for ·OH radical generation. The coexistence of these reactive oxygen species facilitates effective degradation of organic dyes. Therefore, the superior photocatalytic performance of the Ho-doped TiO_2_ samples compared to pure TiO_2_ can be attributed to improved charge carrier dynamics and defect-assisted charge separation rather than major shifts in band edge positions [39,40].

### 3.6. Photoluminescence (PL) Study

Charge transfer, surface defects, and the efficiency of charge carrier trapping have all been investigated using photoluminescence emission spectra. Additionally, PL spectra demonstrated how photo-induced electron-hole pairs behaved in the prepared metal oxide semiconducting material. As shown in the figure, photoluminescence was conducted at room temperature with an excitation wavelength of 320 nm (UV-A light). The reverse radiative deactivation of titanium species caused the electron-hole pairs to recombine after photons were emitted during photocatalysis. The PL spectra indicated that rare earth ion modification reduced peak intensity. Due to energy transfer from titanium ions to Ho^3+^ ions, the intensity of (Ho)-doped TiO_2_ nanoparticles decreased. Defect states were thus created between TiO_2_’s valence and conduction bands [41]. The photocatalyst reduced the recombination rate of electron-hole pairs, enabling the degradation of various organic dyes such as Carmoisine and Crystal Violet.

**Figure 8:**
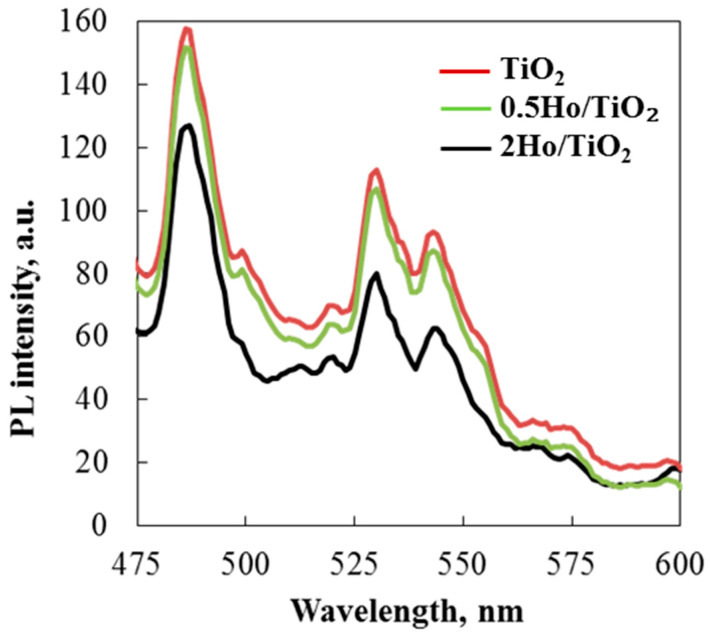
Room temperature of PL spectra of TiO_2_ and 2Ho/TiO_2_ catalysts.

The emission peaks at 486 and 518 nm represent charge transfer from titanium ions to oxygen vacancies (Figure 8). The strongest peaks at 530 and 544 nm represent green emissions attributed to defect electronic states caused by oxygen vacancies and Ho^3+^ ion incorporation in TiO_2_ [42,43]. Photoluminescence emission spectra observed in the green region, between 500 and 570 nm, are due to charge carriers trapped around surface oxygen vacancies [44]. Notably, when Ho is introduced, the PL feature intensity of TiO_2_ decreases. This suggests that the Ho/TiO_2_ powder inhibits photogenerated electron-hole pair recombination, confirming the higher photocatalytic activity of the modified titanium dioxide.

### 3.7. Photocatalytic Efficiency of Pure and Ho Modified TiO_2_ Powders

The photocatalytic activity of Carmoisine (cationic dye) and Crystal Violet (anionic dye) is examined by decomposing them using pure and Ho-doped TiO_2_. To allow the pollutant to adsorb and desorb onto the samples, the dye aqueous solution must first be left in the dark for 30 min. Carmoisine and Crystal Violet decolorization rates are measured spectrophotometrically. Results show that both self-degradation and adsorption are almost nonexistent. This highlights the importance of the catalyst. The catalytic properties of pristine and Ho-modified TiO_2_ are clearly affected by the annealing temperature.

**Figure 9:**
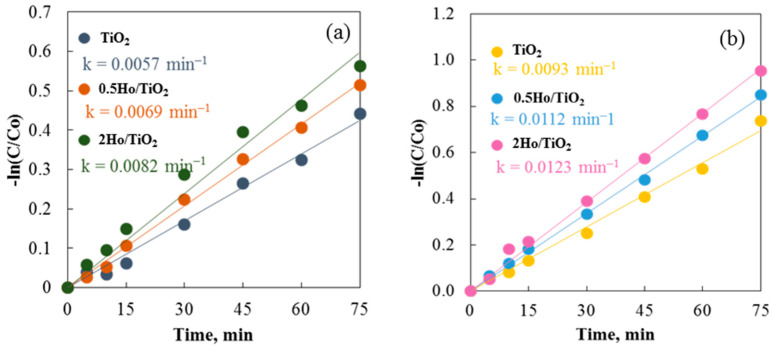
Photocatalysis of Carmoisine using (**a**) non-annealed and (**b**) annealed TiO_2_ and Ho/TiO_2_ powders.

The catalytic efficiency of annealed and non-annealed TiO_2_ and Ho/TiO_2_ powders is displayed in Figure 9. As observed, the annealed samples exhibit significantly better catalytic properties than the non-annealed samples. The annealing process likely promotes the growth of active surfaces. Due to the uniformity and homogeneity of the surface, carrier participation in redox reactions is enhanced [45]. Notable differences exist in the morphology of the two sample types. Annealed powders feature irregularly shaped particles of varying sizes scattered across the surface. Non-annealed catalysts have typical sol–gel materials morphology with agglomerated particles. Agglomeration reduces the active surface area and increases electron-hole recombination probability. As a result, particles that have been annealed break down the cationic dye faster than those that have not. Using both annealed and non-annealed TiO_2_ and Ho/TiO_2_ powders, Figure 9 shows and contrasts the rate constants from all photocatalytic studies. The common pseudo-first-order kinetics equation −Ln(C/C_0_) = kt was utilized to compute the reaction rate constants (k) [46]. These findings are supported by the fact that all annealed catalysts have higher rate constants. Using TiO_2_, 0.5Ho/TiO_2_, and 2Ho/TiO_2_, the degradation efficiencies of the annealed samples were 52.1%, 57.2%, and 61.5%, respectively.

**Figure 10:**
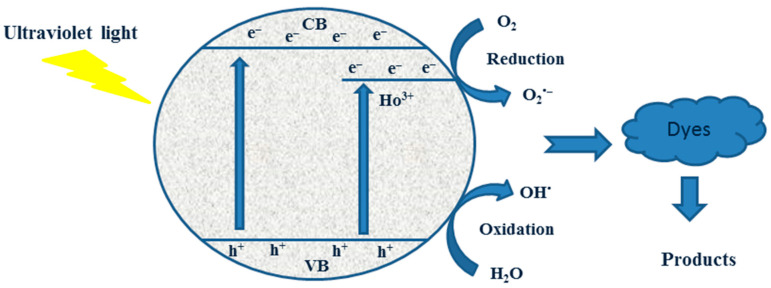
The proposed photocatalytic mechanism shows that photogenerated electron-hole pairs are separated and transferred across Ho/TiO_2_ upon exposure to UV light.

Moreover, the effect of the Ho^3+^ in the catalyst was also examined alongside the annealing temperature. In comparison with pure TiO_2_, Ho^3+^ doping significantly improved TiO_2_’s photocatalytic activity. From Figure 10, it is apparent that the amount of holmium doping played a key role in influencing photocatalytic activity. The photocatalytic reactions took place on the catalyst’s surface, and because the photogenerated electrons and holes responded quickly, interfacial charge carrier transfer was only possible if the donor or acceptor was pre-adsorbed prior to the reaction. Larger surface areas helped stop electron-hole recombination on the catalyst surface by enabling samples to pre-adsorb more Carmoisine molecules. In the TiO_2_ photocatalyst, the presence of Ho^3+^ dopant reduced the crystalline size, which likely increased the number of oxygen vacancies and/or surface defects. Because of the reduced crystallite size, photogenerated carriers were more likely to travel to the surface and react with the substrate. To put it another way, the transfer efficiency of photogenerated carriers to supports and adsorbed compounds on the photocatalyst matrix was probably enhanced by holmium doping, which increased photocatalytic activity. Additionally, as excited electrons and positive holes move toward the surface, holmium doping may stop them from recombining.

This explains the increased activity and quicker breakdown of pollutants. It is known that when Ho^3+^ concentrations are increased from 0.5% to 2%, the band gap energy tends to decrease. The increase in the percentage concentration of Ho^3+^ suggests that the Ho/TiO_2_ semiconductor structure can increase the effectiveness of photoinduced charge separation, which will enhance the sample’s photocatalytic properties [47,48,49]. The computed positions of the 2Ho/TiO_2_ conduction bands have the most negative values (−0.23 to −0.24 V vs. NHE) compared to the values of the other samples. Therefore, this proves the formation of more superoxide radicals, which contribute to the faster degradation of food dyes. On the other hand, substituting Ti ions with Ho ions could cause charge imbalance, which might be offset by increased hydroxide ion adsorption on the surface [50]. These hydroxide ions can trap holes and improve the separation efficiency of electron-hole pairs. Furthermore, hydroxide ions facilitate hydrogen peroxide breakdown of Carmoisine by reacting with holes to generate surface hydroxyl radicals. Thus, holmium-doped TiO_2_’s photocatalytic activity can be enhanced while suppressing charge carrier recombination.

The region of the band gap narrows (Figure 10) as the amount of doping increases, and the strong electric field effectively separated the electron-hole pairs inside the region prior to recombination. At high doped ion concentrations, however, the space charge region narrows, and light penetrates deeper into TiO_2_ than the space charge layer, making it easier for photogenerated electron-hole pairs to recombine in semiconductors (Figure 9).

**Figure 11:**
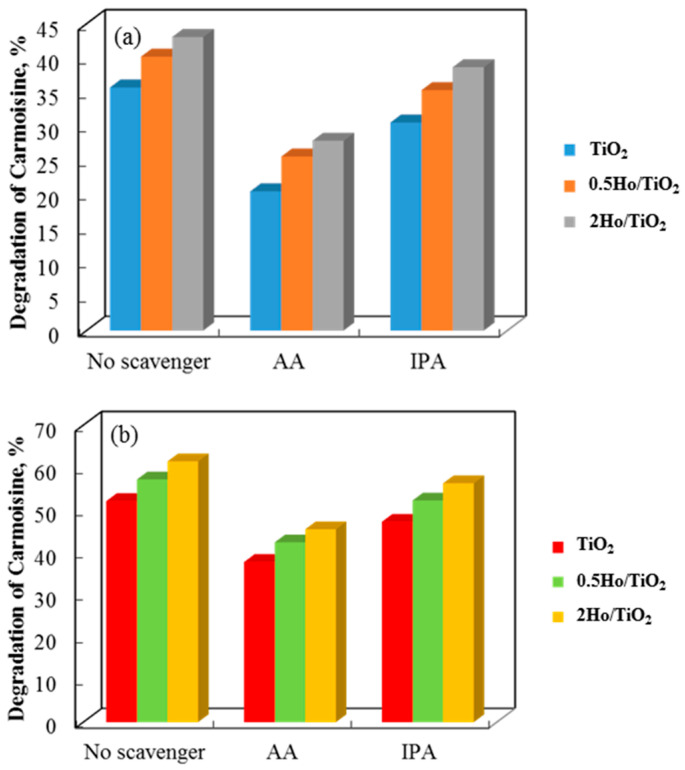

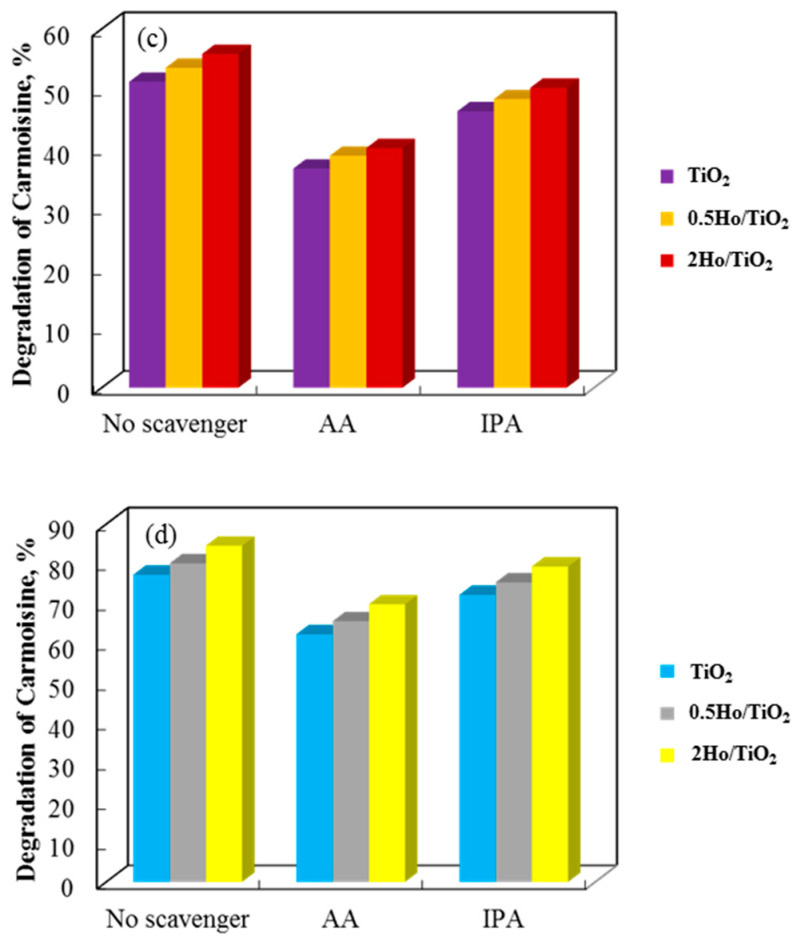
Degradation degree of Carmoisine and Crystal Violet using non-annealed (**a**,**c**) and annealed catalysts (**b**,**d**) in the presence of AA and IPA scavengers.

The effects of adding ascorbic acid (AA) and isopropyl alcohol (IPA) to the three photocatalyst systems are shown in Figure 11, with the former showing a stronger inhibition. The addition of ascorbic acid (AA) and isopropyl alcohol (IPA) scavengers, which capture the corresponding reactive species, clearly quantified the roles of superoxide and hydroxyl radicals in the breakdown of Carmosine and Crystal Violet. This suggests that the super-oxide radical has a greater influence on the photodegradation rates of both dyes.

TiO_2_ has CB and VB potentials of −0.313 eV and 2.93 eV, respectively (Table 1). 2Ho/TiO_2_ has CB and VB potentials of −0.23 eV and 2.85 eV, respectively (Table 1). Theoretically, photogenerated electrons on the TiO_2_ CB migrate to the Ho^3+^ CB, and photogenerated holes on the Ho^3+^ VB migrate to the TiO_2_ VB when TiO_2_ and Ho^3+^ are photoexcited under UV light. This indicates that a II-Type semiconductor heterojunction photoinduced charge-transfer mechanism is used following the coupling of Ho^3+^ and TiO_2_. However, it is evident from the experimental results of active species that the photodegradation process produced ⋅O^2−^ and ⋅OH free radicals. The accumulation of photogenerated holes in the TiO_2_ VB and the production of ⋅O^2−^ free radicals in the Ho^3+^ CB are not facilitated by the coupling of Ho^3+^ and TiO_2_ through II-Type semiconductor junctions. Consequently, the II-Type semiconductor heterojunction process does not transmit the photoinduced charges in the Ho/TiO_2_ composite. Rather, a more appropriate explanation is the creation of semiconductor heterojunctions between Ho^3+^ and TiO_2_. The photogenerated electrons on the Ho^3+^ CB and the photoinduced holes on the TiO_2_ VB are preserved when photogenerated electrons on the TiO_2_ CB immediately recombine with photogenerated holes on the Ho^3+^ VB. This guarantees that the photoinduced charges in the photodegradation mechanism are separated. Concurrently, photogenerated electrons enriched in the Ho^3+^ CB have enough reducing power to produce ⋅O^2−^ radicals, and holes enriched in the TiO_2_ VB have enough oxidation power to produce ⋅OH radicals [51].

**Figure 12:**
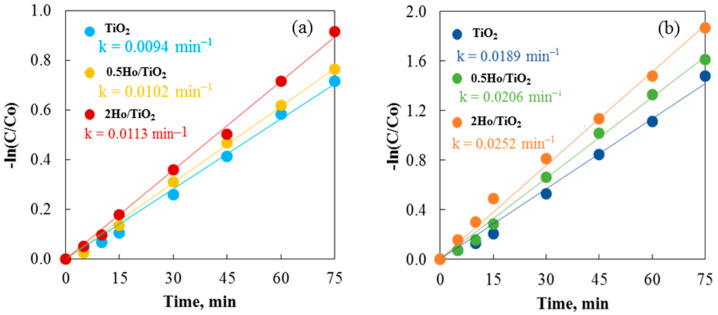
Photocatalysis of Crystal Violet using (**a**) non-annealed and (**b**) annealed TiO_2_ and Ho/TiO_2_ powders.

It is noteworthy that a difference in the activity of the pure and Ho-doped TiO_2_ photocatalysts for cationic and anionic dyes was found. Figure 12a,b show how non-annealed and annealed TiO_2_ powders remove Crystal Violet and Carmoisine in 75 min. Carmoisine took longer to remove than Crystal Violet, which had better removal results. The rate constant values for both dye removals were calculated using the slopes of the straight lines that result from plotting ln(C/C_0_) against illumination time. A larger rate constant was observed when comparing the elimination of crystal violet to that of Carmoisine. Carmosine, a cationic dye, has a positive charge, while Crystal Violet, an anionic dye, has a negative charge. Photosensitization with cationic dye is very difficult due to the positive charge of the TiO_2_ surface and the associated electrostatic repulsion between the dye molecules and the TiO_2_ surface [52]. The reduced removal capacity of both annealed and non-annealed TiO_2_ and Ho/TiO_2_ is thus explained. This idea is supported by Hasnat and associates [53]. UV light irradiation enhanced the oxidation of adsorbed dye molecules on the surface of TiO_2_ particles, potentially speeding up the removal process. Non-annealed 2Ho/TiO_2_ may efficiently remove Carmoisine, achieving around 61% elimination in 75 min. It is clear that if the removal process is allowed to go on for a longer amount of time, holmium-modified TiO_2_ may remove more dye. However, by attaining 85% removal at the same time, annealed 2Ho/TiO_2_ demonstrates its prominence in removing Crystal Violet. It demonstrated once more that TiO_2_’s positive surface charge made it effective at eliminating anionic dye.

**Figure 13:**
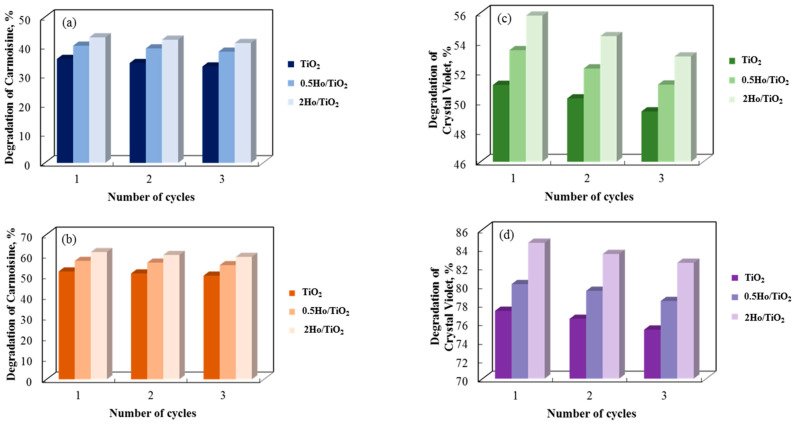
Carmoisine and Crystal Violet decolorization rate in the presence of non-annealed (**a**,**c**) and annealed (**b**,**d**) catalysts for three consecutive cycles.

A three-cycle investigation on the recyclability of annealed and non-annealed photocatalysts made of pure and holmium-modified TiO_2_ is presented in Figure 13. After three cycles in distilled water, the photocatalytic breakdown of Carmoisine and Crystal Violet decreased by roughly 2% for all catalyst types, indicating a small deterioration in the photocatalysts’ catalytic efficacy with each cycle. Despite this decrease, the sol–gel samples’ dye degradation cycle was determined to be steady. These results demonstrate that they can be used repeatedly to degrade paracetamol. Ho^3+^ is the most stable and efficient catalyst over numerous cycles, despite 2Ho/TiO_2_ showing a modest decline with repeated usage.

**Figure 14:**
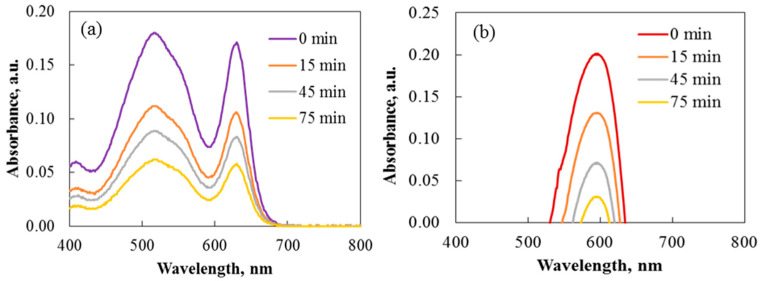
Absorbance spectra of the degradation of Carmoisine (**a**) and Crystal Violet (**b**) under ultraviolet illumination using annealed 2Ho/TiO_2_ powders.

The photocatalytic effectiveness of rare-earth-modified TiO_2_ powder was studied for the decomposition of Carmoisine and Crystal Violet under UV light. The process was supported by magnetic stirring, and each experiment kept the dye concentration at 7.5 mg/L. UV/Vis spectroscopy tracks the absorption peaks of the dyes to monitor their degradation. Spectral changes during the breakdown of Carmoisine and Crystal Violet were examined to determine how Ho^3+^ affects TiO_2_ activity throughout the photocatalytic process. The UV/Vis spectra for dye degradation using pure 2Ho/TiO_2_ are shown in Figure 14.

## 4. Conclusions

The sol–gel process was successfully used to create Ho-modiffied TiO_2_ nanoparticles. Since the XRD patterns showed no distinct holmium oxide peaks, the SEM-EDS verified that Ho had been successfully incorporated into TiO_2_ anatase. The optical characteristics of the examined samples were not significantly affected by the Ho^3+^ doping, according to UV-vis absorption studies. The photocatalytic characteristics of samples annealed at 500 °C are superior to those of non-annealed samples. The combined semiconductor Ho/TiO_2_ is shown to have better photodegradation efficiency of organic pollutants than pure titanium dioxide. The 2Ho/TiO_2_ sample has the best photocatalytic properties regardless of the dye structure. The semiconductors have higher degradation efficiency for anionic dyes.

## Figures and Tables

**Table 1:** Energy of the band gap, potentials of current band (CB) and valence band (VB), cut-off.

Sample	*E_g_*, eV	*E_CB_*, eV	*E_VB_*, eV	Cut-Off, nm	Reference
2Ho/TiO_2_	3.08	−0.230	2.85	402.5	This paper
0.5Ho/TiO_2_	3.10	−0.238	2.86	400	This paper
TiO_2_	3.25	−0.313	2.93	380.44	[23]

## Data Availability

The original contributions presented in this study are included in the article. Further inquiries can be directed to the corresponding authors.
